# Beneficial effects of whey protein peptides on muscle loss in aging mice models

**DOI:** 10.3389/fnut.2022.897821

**Published:** 2022-09-09

**Authors:** Xin Wu, Xiaochen Yu, Na Zhu, Meihong Xu, Yong Li

**Affiliations:** ^1^Department of Nutrition and Food Hygiene, School of Public Health, Peking University, Beijing, China; ^2^Beijing Key Laboratory of Protein Posttranslational Modifications and Cell Function, Department of Biochemistry and Molecular Biology, School of Basic Medical Science, Peking University Research Center on Aging, Peking University, Beijing, China

**Keywords:** whey protein peptides, d-galactose (d-gal), sarcopenia, age-related, skeletal muscles, protein synthesis, muscle loss

## Abstract

Aging-related muscle loss is a hallmark of aging and is the cause of some negative outcomes. An optimized diet and supplements have a positive effect in slowing down the process of muscle loss. D-galactose(d-gal) has been used widely to develop aging model. This study explored the beneficial effects of whey protein peptides (WPPs) on sarcopenia in d-gal-induced aging mice. A total of 72 SPF male C57BL/6N mice were used in this study. Sixty mice were modeled by injected intraperitoneally with d-gal (100 mg/kg body weight for 6 weeks), and the other 12 mice were used as control, and injected with the same amount of normal saline. After 6 weeks, the modeled mice were randomly divided into the model control group, whey protein group (1.5 g/kg^*^bw), and three WPPs intervention groups (0.3 g/kg^*^bw, 1.5 g/kg^*^bw, 3.0 g/kg^*^bw), according to serum malondialdehyde (MDA) level. The test samples were orally given to mice by daily garaged. During the 30 days intervention period, the model control group, whey protein group, and WPPs group continued receiving intraperitoneal injections of d-gal, whereas the control group continued receiving intraperitoneal injections of normal saline. The results showed that WPPs could significantly improve the grip strength of aged mice. WPPs could significantly increase lean mass of aged mice and increase muscle weight of gastrocnemius and extensor digitorum longus. WPPs could significantly increase the level of insulin-like growth factor-1 (IGF-1) and reduce level of interleukin (IL)-1, IL-6, tumor necrosis factor-alpha (TNF-α) in serum. WPPs could affect the muscle fiber size in d-gal-induced aging mice. Its specific mechanism may be related to the activation of IGF-1/Akt/mTOR protein synthesis signaling pathway and reduction of the level of inflammation. These results indicate that WPPs can improve aging-related sarcopenia. Compared with whey protein, WPPs supplement seems a better form for sarcopenia.

## Introduction

With the increasing life expectancy, the world is experiencing a dramatic aging process. According to World Health Organization (WHO) data, the number of elderly people (aged 60 years and above) is expected to exceed 1.4 billion in 2030 and then 2.1 billion in 2050. For individuals, aging leads to degenerative changes occurring at multiple levels, from molecular metabolism to organ systems, which contributed to various health problems (1). As the largest motor organ in the body, skeletal muscle loses its mass, size, strength, and function, presenting an age-related hypofunction. The age-related hypofunction of skeletal muscles compromises the physical and social functional abilities of the elderly. Moreover, defined as a progressive and generalized skeletal muscle frailty, sarcopenia [*International Classification of Diseases, Tenth Revision, Clinical Modification* (ICD-10-CM)] is closely related to an increased likelihood of adverse consequences such as physical disability, falls, fractures, and even mortality (2–4). Being intended to enable earlier interventions, the Asian Working Group for Sarcopenia (AWGS) 2019 introduced a “possible sarcopenia” conception (5). According to this diagnostic criterion, the prevalence of possible sarcopenia, sarcopenia, and severe sarcopenia was 38.5, 18.6, and 8.0%, respectively, among the older Chinese population (6). Globally, the numbers must be terrible. All these point to a huge personal and social burden. However, due to the unclear pathophysiology of sarcopenia, there are no available agents for muscle building currently approved worldwide. Nutritional supplementation and resistance training are still the primary strategies in both prevention and treatment of sarcopenia across the life course (7).

A large number of studies demonstrated that if older adults with sarcopenia consumed sufficient dietary protein, then the muscle mass and physical performance would be improved, either by ordinary food intake or by supplementation (8–10). Whey proteins contain all essential amino acids and are easily digestible (11). With various bioactive components, such as branched-chain amino acids (BCAA) and bioactive peptides, the whey proteins have been widely used in clinical adjuvant therapy, nutritional intervention, and some other healthcare fields (11, 12). Whey protein supplements should be a good choice for the elderly, especially those with weak absorption capacity. Accumulating evidence (13) has shown that whey protein intervention can delay or reduce symptoms of sarcopenia and improve biomarker disorder, based on clinical trials (14–17), animal experiments (18), and cell cultures (19, 20).

Peptide is a relatively low-molecular-weight hydrolytic form of protein. Previous studies have shown that bioactive peptides have the advantages of high absorption and utilization rate than protein, whereas they do not typically cause adverse events. Moreover, it has been shown that bioactive peptides have health-promoting benefits and biological activities (21). Therefore, protein-derived bioactive substances can not only meet the protein intake of older adults but also produce more bioactive effects for the elderly. Whey protein peptides (WPPs) are a group of active peptides produced from whey protein by enzymatic hydrolysis. As a typical kind of bioactive peptides, WPPs have anti-inflammatory, antioxidative, immunological, and some other biological activities, regulating blood lipids and improving learning and memory in elderly (22–24). However, less research has been done on muscle loss and sarcopenia, especially on the beneficial effects of WPPs. To the best of our knowledge, these experiments were mainly carried out in muscle cells lines (25, 26), which were barely in aged animals. Thus, whether these peptides can get through the gut barrier and be effective in the body? Peptide or protein, which is the best form of supplement for sarcopenia patients? These questions are unknown.

D-gal-induced aging animal models have been extensively used to study aging mechanisms and anti-aging effects of drugs (27). Many studies show that the accumulation of d-gal alters the antioxidant system and speeds up the process of aging (28). The previous studies showed that d-gal induced model could be a good choice for the study of age-dependent skeletal muscle degeneration (29, 30). Therefore, we applied d-gal-induced aging mice model to simulate the skeletal muscle aging. Hence, this study sought to assess the beneficial effect of WPPs on muscle loss in d-gal-induced aging mice and explored the possible mechanisms of the role of WPPs. Specifically, we added a whey protein group to validate the differences between peptides and protein. The study aimed to provide direct evidence of optimal supplemental form for sarcopenia.

## Materials and methods

### Preparation of WPPs

#### Whey protein peptides

A milky white solid powder was extracted from the proteins of whey *via* enzymatic hydrolysis and provided by Tianjin Milkyway Import and Export Co., Ltd., Tianjin, China. The WPPs are a small-molecule bioactive peptide mixture, which is separated from whey protein using enzymatic hydrolysis technology. WPP8350 produced by Hilmar was used in this study. The protein peptide content of WPP8350 was 67.2%, which contains more glutamic acid, aspartic acid, and leucine. The detailed amino acid composition of WPPs is shown in Table 1.

**Table 1:** Amino acid composition of WPPs.

**Amino acid**	**Content (g/100 g)**
Glu	14.4
Val	4.6
Thr	5.8
Asp	8.9
Ile	5.4
Ala	4.3
Leu	8.6
Ser	4.2
Arg	2.3
Trp	1.7
Hyp	<0.1
Gly	1.5
Phe	2.6
Lys	8.1
His	1.5
Met	1.8
Pro	5
Tyr	2.5
Cys	2

Glu, glutamic acid; Val, valine; Thr, threonine; Asp, Aspartic acid; Ile, isoleucine; Ala, alanine; Leu, leucine; Ser, serine; Arg, argnine; Trp, tryptophane; Hyp, hydroxyproline; Gly, glycine; Phe, phenylalanine; Lys, lysine; His, histidine; Met, methionine; Pro, proline; Tyr, tyrosine; Cys, cysteine. Whey protein and WPP were obtained from Tianjin Milkyway Import and Export Co., Ltd. (Tianjin, China).

### Animals

A total of 72 healthy SPF male C57BL/6N mice (weight 40 ± 10 g, 6 months) provided by the Peking University Health Science Center were used in this study. The mice were kept in the SPF animal house, at the temperature range of 22 ± 2°C, relative humidity of 50%~60%, and time between day and night of 12 h:12 h. Two mice were housed in a cage and they ate freely during the experiment. All experiments were approved by the Peking University Animal Research Committee. Experimental Animal Production License No. SCXK (Beijing) 2016-0010; Experimental Animal Use License No. SYXK (Beijing) 2016-0041. All animals were treated according to the principles of laboratory animal care and guidelines of the Peking University Animal Research Committee.

### Experimental design

A total of 12 mice were randomly selected as the normal control group after a period of adaptive feeding. All mice, except the control group, were injected intraperitoneally with d-gal, 100 mg/kg body weight, at a dose of 0.1 ml/10 g for 6 weeks. The mice in the control group were injected intraperitoneally with the same amount of normal saline once a day for 6 weeks. After 6 weeks, blood samples were collected from the tail vein and centrifuged at 3,000 rpm for 10 min to separate the serum. Serum MDA content was detected and the mice were then randomly divided into two groups according to the level of MDA. After grouping, no significant difference was found in the MDA level between model group (*p* > 0.05), but there was a significant difference in the MDA level between the model group and control group (*p* < 0.05).

After the model was established, the mice were randomly divided into the following five groups according to MDA level: one model control group, one whey protein group (1.5 g/kg ^*^body weight), and WPPs low-, medium-, and high-dose group (0.3, 1.5, and 3.0 g/kg ^*^body weight, respectively, referred to as WPPs-LG, WPPs-MG, and WPPs-HG). The dose was set according to the pre-experiment and the results of our research group's previous whey protein peptide experiments (23). All mice were administered by gavage every day, the mice in the normal control group and model control group were given high-pressure sterilized distilled water, the whey protein group and WPPs group were given corresponding concentrations of the test substance, and WPPs and whey protein solution were prepared with distilled water. At the same time, the mice in the model control group, whey protein group, and each dose group were given the same dose of d-gal intraperitoneally, while the mice in the control group was administered intraperitoneal injections of normal saline. The animals were weighed twice a week, and the sample dose was adjusted according to body weight. The test samples were given for 30 consecutive days. During the experiment, the general conditions of mice in each group were observed weekly, including food intake, hair removal, weakness, and physical activity, etc.

### Analysis of physical performance tests and body composition

The age-dependent physical performance of C57BL/6N mice was tested, including grip strength and wire hang. We used forearm grip strength to measure muscle strength in the grip strength test and suspension force was tested by the wire hang test. During the grip strength test, each mouse grabbed a grip meter with its forelimb and gradually pulled back until they released their grip. The maximum force was recorded in gram (g). The tests were repeated five times. The maximum among the five times was recorded as the grip strength of individual mouse. In the wire hang test, the mouse was placed onto the top of a wire mesh cage, which was then gently inverted to encourage the mouse to grip the wire. The retention time was recorded. We set the maximum duration at 120 seconds. The average of the two times was recorded as the individual mouse muscle strength. To estimate the ratio of the fat mass and the muscle mass at the total body level, the body composition detection using EchoMRI was performed according to the manufacture's instruction.

### Tissue collection

After the examination, the mice were sacrificed by dislocation of cervical vertebra. After the mice were killed, the muscle was separated immediately. The gastrocnemius (GAS), extensor digitorum longus (EDL), tibialis anterior (TA) and soleus muscles (SOL) were excised from both hindlimbs. The gastrocnemius from left leg was frozen by liquid nitrogen in Tissue-Tek OCT for immunofluorescence staining and imaging, and the other muscles of left leg were frozen in liquid nitrogen immediately. The muscles from the right leg were weighed, and frozen in liquid nitrogen. All tissue samples were stored at −80°C until analysis. A part of these tissues was used for western blot analysis.

### Enzyme-linked immunosorbent assay

At the end of the experiment, the mice were sacrificed by eyeball blood sampling and cervical dislocation. The level of serum biomarker [insulin-like growth factor-1 (IGF-1), interleukin (IL)-1, IL-6, tumor necrosis factor-alpha (TNF-α)] was determined by assay kits, according to the protocol provided by the manufacturer.

### Immunofluorescence staining and imaging

In order to perform immunofluorescence staining for muscle sections, we collected mouse GAS specimens, froze them with liquid nitrogen in tissue-Tek OCT, and then sliced the muscle sections into 20um with Cryostat (CM1950, Leica). The muscle sections were fixed with paraformaldehyde PFA (4%, 15 min), washed by PBS 3 times, infiltrated by Triton x-100 (0.1%, 10 min) and blocked by Mouse on Mouse (M.O.M.). Block reagent (carrier laboratory) and 5% BSA/5% normal goat serum /PBS were incubated with primary antibody. Antibodies used included mouse anti-MyHC I (BA-D5-S 1:100, DSHB), mouse anti-MyHC IIb (BF-F3 1:100, DSHB) and rabbit anti-laminin (AB2034 1:500, Millipore). Sections were rinsed with PBS/0.1% Tween-20 and incubated with Alexa Fluor 488 or Alexa Fluor 568 labeled secondary antibody (Invitrogen, 1:800). Slides were imaged on LEICA TCS SP8 (LEICA, Germany) confocal microscope.

### Western blot analysis

The expression of phosphop-Akt (Ser473), Akt, phospho-p70S6K (Thr389), p70S6K, phosphop- mTOR (Ser2448), mTOR proteins in muscle tissue of the experiment mouse were determined by Western blot analysis. Antibodies against phosphop-Akt (Ser473) and phospho-p70S6K (Thr389) were from Cell Signaling (Beverly, MA, USA). phosphop-mTOR (Ser2448) and mTOR antibody were from Abcam. Akt and p70S6K antibodies were from Proteintech Antibody.

### Statistical analyses

We used SPSS software version 22 (SPSS Inc., Chicago, IL, USA) for statistical analyses. The values were presented as mean ± standard deviation (SD). Differences between groups were analyzed by a one-way analysis of variance test and LSD methods if the data were homogeneous, or the Dunnetts'T3 test if variances were unequal.

## Results

### Effects of WPPs on body and muscle weights in mice

**Figure 1:**
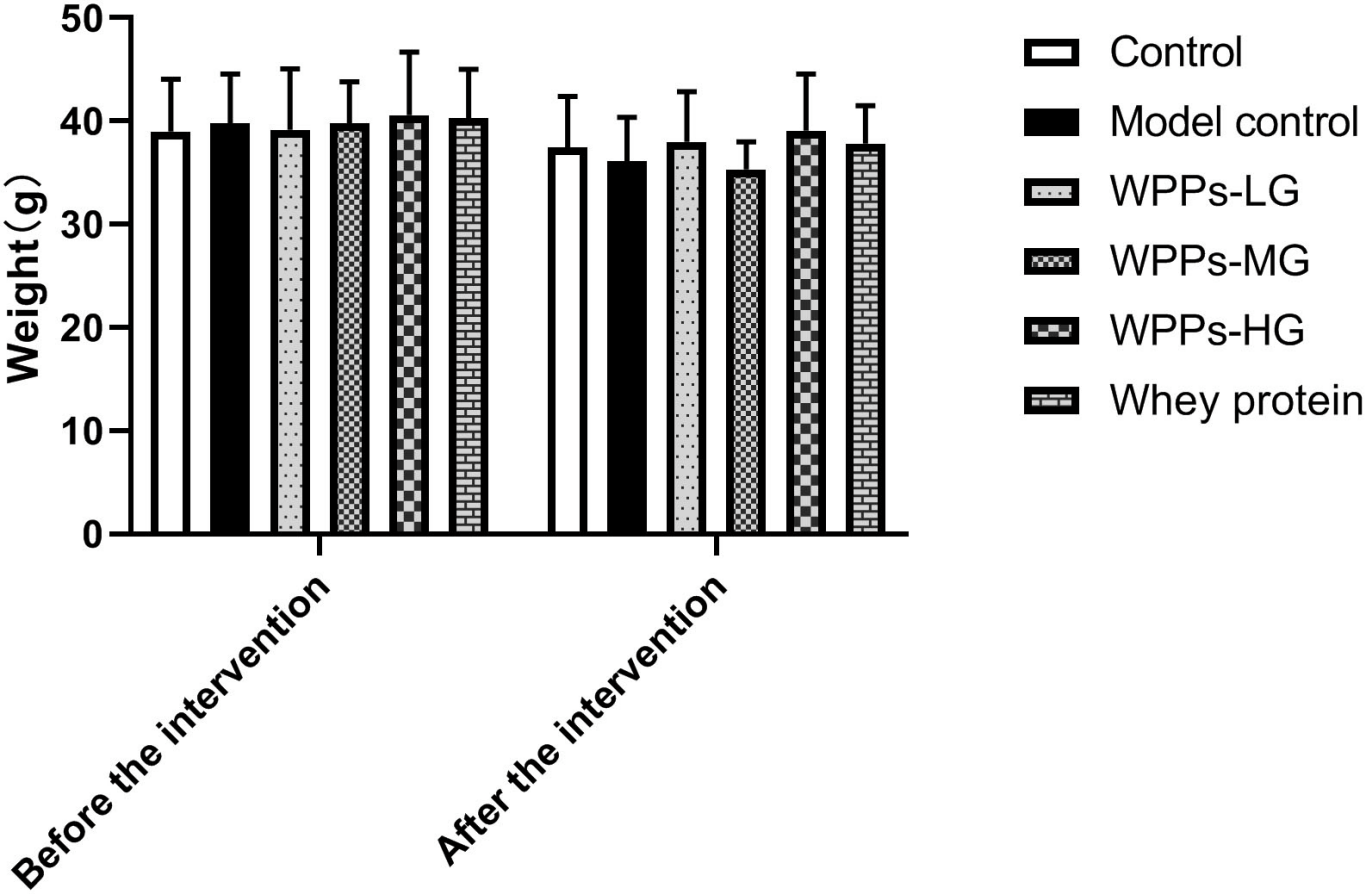
Effects of WPPs on body weight. Data are expressed as means ± SD (*n* = 12). Whey protein group at a dose of 1.5 g/kg; WPPs-LG, whey protein peptides low-dose group at a dose of 0.3 g/kg; WPPs-MG, whey protein peptides medium dose group at a dose of 1.5 g/kg; WPPs-HG, whey protein peptides high-dose group at a dose of 3.0 g/kg.

We measured the body weight of mice before the intervention (beginning of WPPs treatment) and after that (finishing of WPPs treatment). There was no significant difference in the body weight among groups before or after intervention (Figure 1). The muscle wet weight of mice in each group is shown in Table 2. Compared with the control group, the GAS and EDL mass in the model control group were both significantly decreased (p < 0.05). The mice in the control group and WPPs-LG groups had a relatively larger muscle mass of GAS than that in the model control group (p < 0.05). Compared with the model control group, the EDL mass in the control group, WPPs-LG, WPPs-MG, and WPPs-HG significantly increased (p < 0.05). However, there was no significant difference for SOL and TA among groups (p > 0.05). As shown Table 2, A marginal increase in EDL muscle weight/ body weight was also observed in WPPs groups. GAS and TA muscle/body weight appeared to be heavier in the WPPs group than in the model control group, although the differences were not significant (Table 2).

**Table 2:** Effects of WPPs on muscle wet weight and muscle weight/BW.

**Groups**	**GAS(mg)**	**SOL(mg)**	**TA(mg)**	**EDL(mg)**	**GAS/BW (%)**	**SOL/BW (%)**	**TA/BW (%)**	**EDL/BW (%)**
Control	157.78 ± 9.83^b^	9.78 ± 3.03	60.84 ± 6.56	20.54 ± 5.11^b^	0.46 ± 0.06	0.03 ± 0.01	0.18 ± 0.03	0.06 ± 0.01^b^^c^
Model control	140.86 ± 10.26^a^	8.91 ± 3.79	55.5 ± 9.43	12.13 ± 2.84^a^	0.40 ± 0.06	0.03 ± 0.01	0.15 ± 0.02	0.03 ± 0.01^a^
WPPs-LG	156.60 ± 4.17^b^	9.73 ± 3.28	63.36 ± 10.95	18.83 ± 6.66^b^	0.43 ± 0.04	0.03 ± 0.01	0.17 ± 0.03	0.05 ± 0.02^b^
WPPs-MG	153.76 ± 14.70	9.38 ± 7.57	58.38 ± 11.51	18.28 ± 6.46^b^	0.44 ± 0.05	0.03 ± 0.02	0.17 ± 0.04	0.05 ± 0.02^b^
WPPs-HG	152.1 ± 11.26	9.14 ± 4.27	55.22 ± 8.061	19.61 ± 6.22^b^	0.40 ± 0.06	0.02 ± 0.01	0.14 ± 0.03	0.05 ± 0.02^b^
Whey protein	155.98 ± 10.88	8.58 ± 2.69	60.63 ± 8.17	15.71 ± 6.36	0.42 ± 0.06	0.02 ± 0.01	0.16 ± 0.02	0.04 ± 0.02^a^

Muscle mass and muscle weight/body weight (BW) for gastrocnemius (GAS), soleus (SOL), tibialis anterior (TA), and extensor digitorum longus (EDL) was are expressed as means ± SD (*n* = 12).

a *p* < 0.05 indicates significant difference versus the control group;

b *p* < 0.05 indicates significant difference versus the model group;

c *p* < 0.05 indicates significant difference versus the whey protein group. Whey protein group at a dose of 1.5 g/kg; WPPs-LG, whey protein peptides low dose group at a dose of 0.3 g/kg; WPPs-MG, whey protein peptides medium dose group at a dose of 1.5 g/kg; WPPs-HG, whey protein peptides high dose group at a dose of 3.0 g/kg.

### Effects of WPPs on body composition

**Figure 2:**
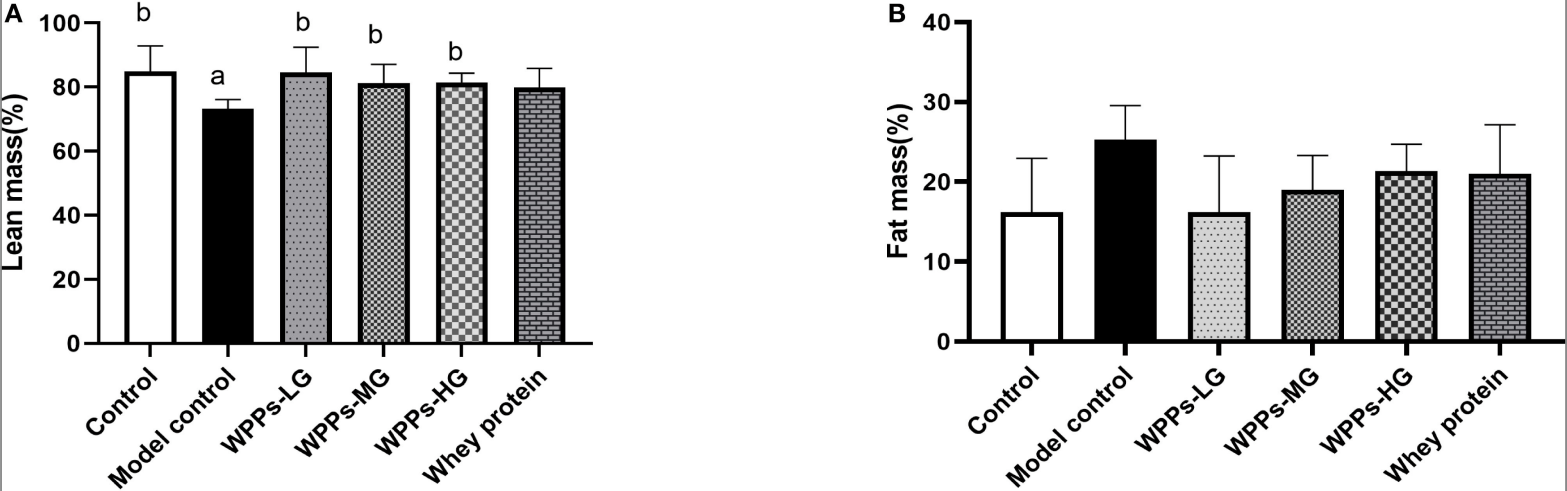
Effects of WPPs on body composition including lean mass **(A)** and fat mass **(B)**. Data are expressed as means ± SD (*n* = 6). ^a^ *p* < 0.05 indicates significant difference vs. the control group; ^b^ *p* < 0.05 indicates significant difference vs. the model group. Whey protein group at a dose of 1.5 g/kg; WPPs-LG, whey protein peptides low-dose group at a dose of 0.3 g/kg; WPPs-MG, whey protein peptides medium-dose group at a dose of 1.5 g/kg; WPPs-HG, whey protein peptides high-dose group at a dose of 3.0 g/kg.

It should be noted that numerous studies examined body composition was very important indicator for diagnosing sarcopenia. Therefore, we studied the difference among groups on body composition. As shown in Figures 2A,B, we measured the body composition including lean and fat. We demonstrate that with the control group, lean mass in the model group significantly decreased (*p* < 0.05). Compared with the model control group, the lean mass in the control group, WPPs-LG, WPPs-MG, and WPPs-HG significantly increased (*p* < 0.05). However, there were no significant differences in fat mass between groups (*p* > 0.05). It suggested that WPPs could increase the lean of d-gal-induced aging mice.

### Effects of WPPs on the physical performance

**Figure 3:**
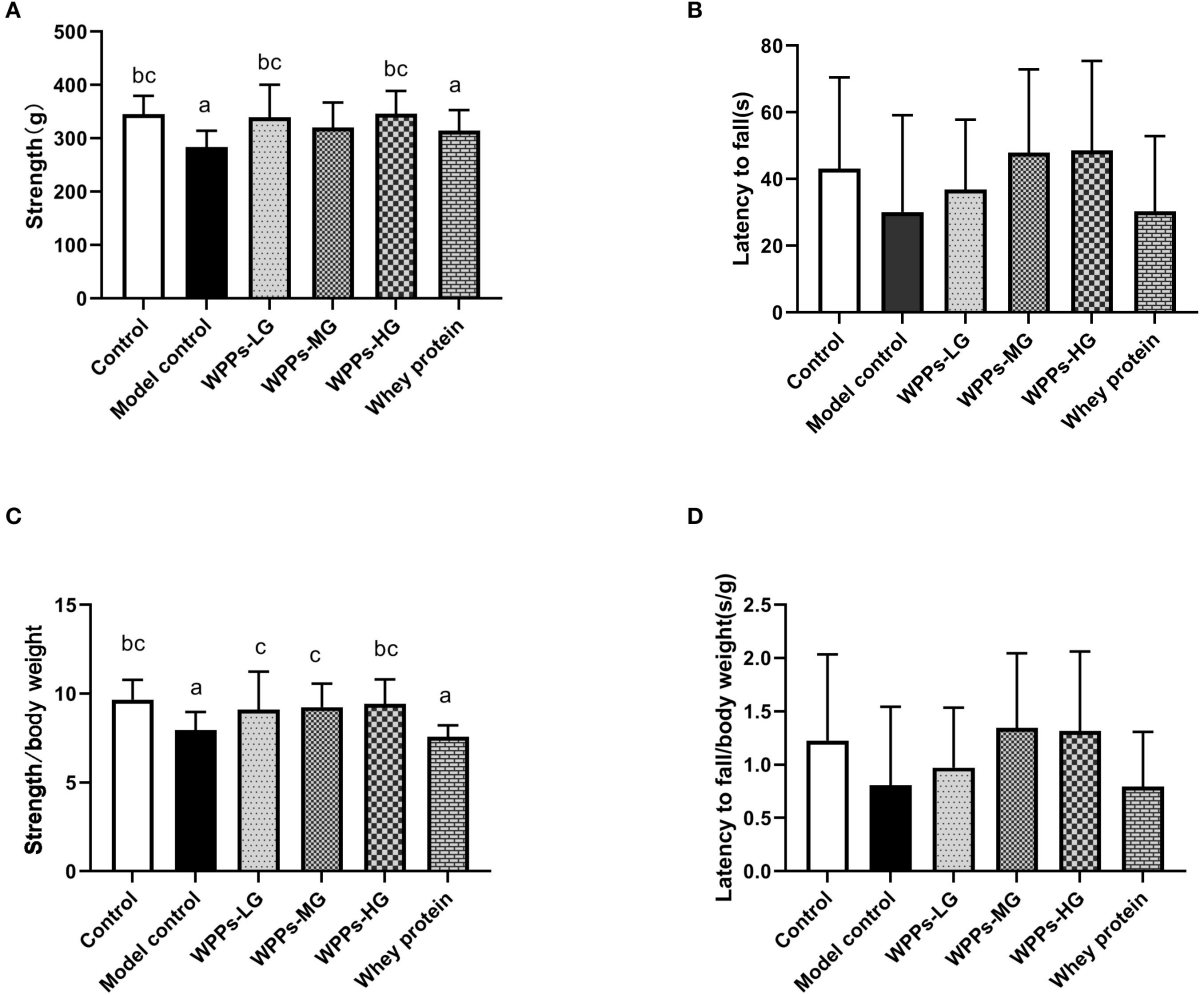
Effects of WPPs on the grip strength **(A)**, suspension force **(B)**, grip strength/body weight **(C)**, suspension force/body weight **(D)**. Data are expressed as means ± SD (*n* = 8). ^a^ *p* < 0.05 indicates significant difference vs. the control group; ^b^ *p* < 0.05 indicates significant difference vs. the model group. ^c^ *p* < 0.05 indicates significant difference vs. the whey protein group. Whey protein group at a dose of 1.5 g/kg; WPPs-LG, whey protein peptides low-dose group at a dose of 0.3 g/kg; WPPs-MG, whey protein peptides medium-dose group at a dose of 1.5 g/kg; WPPs-HG, whey protein peptides high-dose group at a dose of 3.0 g/kg.

In the grip strength test, the maximum grip strength of mice in the model control and the whey protein group was markedly decreased than that in the control group (*p* < 0.05). Compared with the model control group and whey protein group, the muscle strength of mice in the control group, WPPs-LG, and WPPs-HG significantly increased (*p* < 0.05) (Figure 3A). However, there were no significant differences between groups in the wire hang test (*p* > 0.05) (Figure 3B). In order to test whether there is still a difference in the grip strength when normalized to body weight, we measured the value of grip strength/ body weight. We found the value of grip strength/ body weight in the control group and WPPs-HG group mice significantly increased than that the model control mice. Moreover, compared with whey protein group, the value of grip strength/ body weight in WPPs group is higher (Figure 3C). Although there were no sensible differences in the time of latency to fall and latency to fall/body weight between WPPs group and model control group, it can be found that the mice in the WPP group fall later than that in the whey protein and model control group Figures 3B,D.

### Effects of WPPs on the IGF-1 in the serum

**Figure 4:**
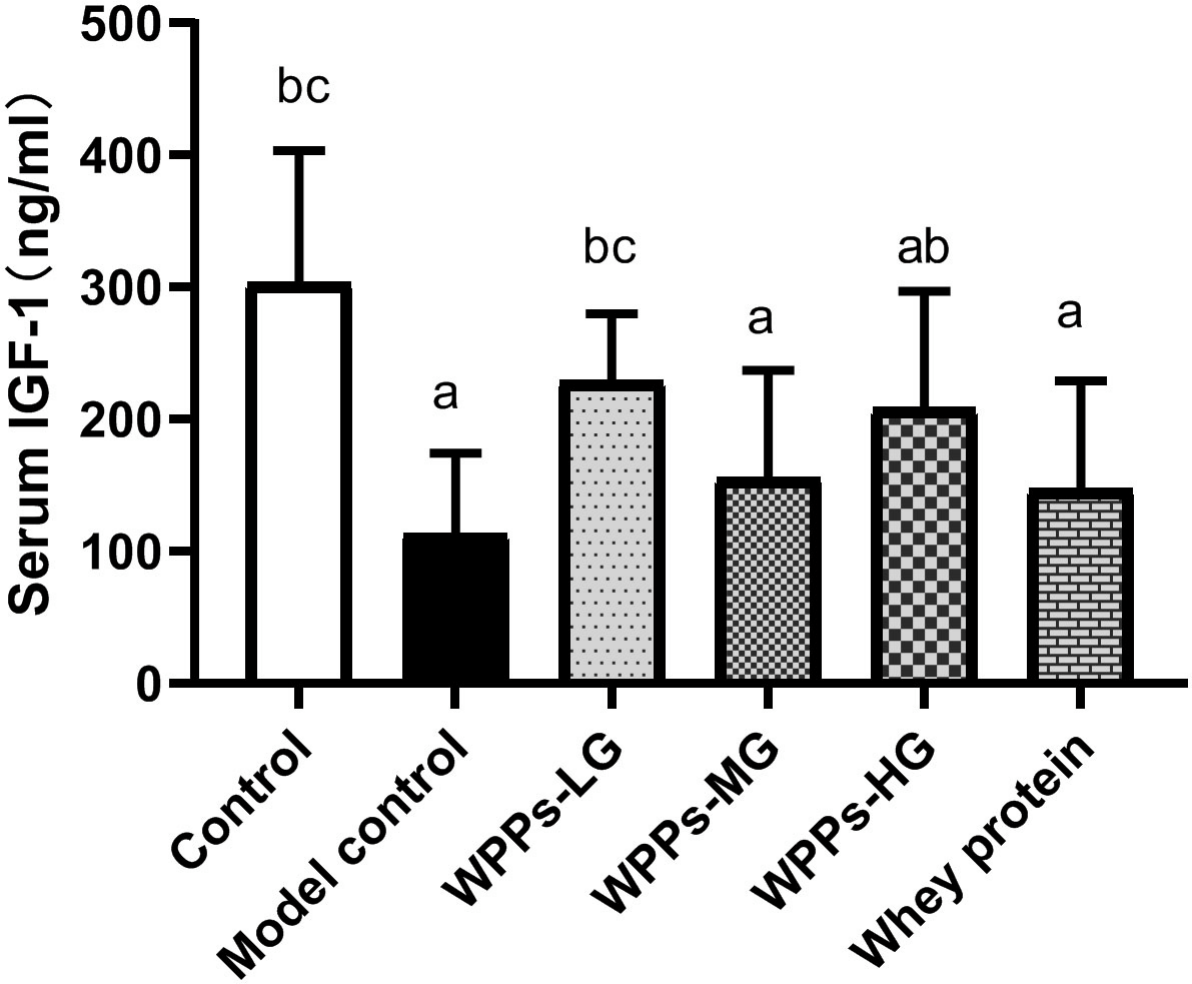
Effects of WPPs on serum IGF-1. Data are expressed as means ± SD (*n* = 8). ^a^ *p* < 0.05 indicates significant difference vs. the control group; ^b^ *p* < 0.05 indicates significant difference vs. the model group; ^c^ *p* < 0.05 indicates significant difference vs. the whey protein group. Whey protein group at a dose of 1.5 g/kg; WPPs-LG, whey protein peptides low-dose group at a dose of 0.3 g/kg; WPPs-MG, whey protein peptides medium-dose group at a dose of 1.5 g/kg; WPPs-HG, whey protein peptides high-dose group at a dose of 3.0 g/kg.

To identify the effects of WPPs on the IGF-1 in the serum, the level of IGF-1 was determined. As shown in Figure 4, we demonstrated that the level of IGF-1 in serum of the model control, WPPs-MG, WPPs-HG, and whey protein group significantly decreased compared to the control group (*p* < 0.05). Compared with the model control group, the level of IGF-1 in the control group, WPPs-LG, and WPPs-HG significantly increased (*p* < 0.05). The result suggests that WPPs can improve the IGF-1 level in the serum of d-gal-induced aging mice.

### Effects of WPPs on the sarcopenia-related inflammatory biomarkers of mice

To further explore whether WPPs could have effect on the sarcopenia-related inflammatory biomarkers, associated inflammatory factors were detected. As shown in Table 3, after administration with WPPs, IL-1β, IL-6, and TNF-α levels in serum were significantly reduced (*p* < 0.05). Additionally, in comparison to the whey protein group, the level of IL-6 in serum in the control group, WPPs-LG, WPPs-MG, and WPPs-HG were significantly reduced (*p* < 0.05). It showed that WPPs had anti-sarcopenia-related inflammatory effects.

**Table 3:** Effects of WPPs on the inflammatory biomarkers.

**Groups**	**IL-1β** **(pg/ml)**	**IL-6** **(pg/ml)**	**TNF-α** **(pg/ml)**
Control	2.61 ± 0.65^b^	51.43 ± 31.54^b^^c^	19.07 ± 5.98^b^
Model control	4.83 ± 2.32^a^^c^	404.94 ± 94.78^a^^c^	42.40 ± 16.84^a^^c^
WPPs-LG	2.24 ± 1.13^b^	75.57 ± 53.42^b^^c^	33.15 ± 13.53^b^
WPPs-MG	2.03 ± 0.98^b^	170 ± 42.87^a^^b^^c^	22.27 ± 17.65^b^
WPPs-HG	2.36 ± 0.67^b^	131.85 ± 56.51^a^^b^^c^	24.84 ± 11.02^b^
Whey protein	2.55 ± 1.34^b^	308.26 ± 63.52^a^^b^	26.57 ± 5.70

Data are expressed as means ± SD (n=6).

a p < 0.05 indicates significant difference vs. the control group;

b p < 0.05 indicates significant difference vs. the model group.

c p < 0.05 indicates significant difference vs. the whey protein group. Whey protein group at a dose of 1.5 g/kg; WPPs-LG, whey protein peptides low-dose group at a dose of 0.3 g/kg; WPPs-MG, whey protein peptides medium-dose group at a dose of 1.5 g/kg; WPPs-HG, whey protein peptides high-dose group at a dose of 3.0 g/kg.

### Effects of WPPs on the effect of cross-sectional area of muscle fibers

**Figure 5:**
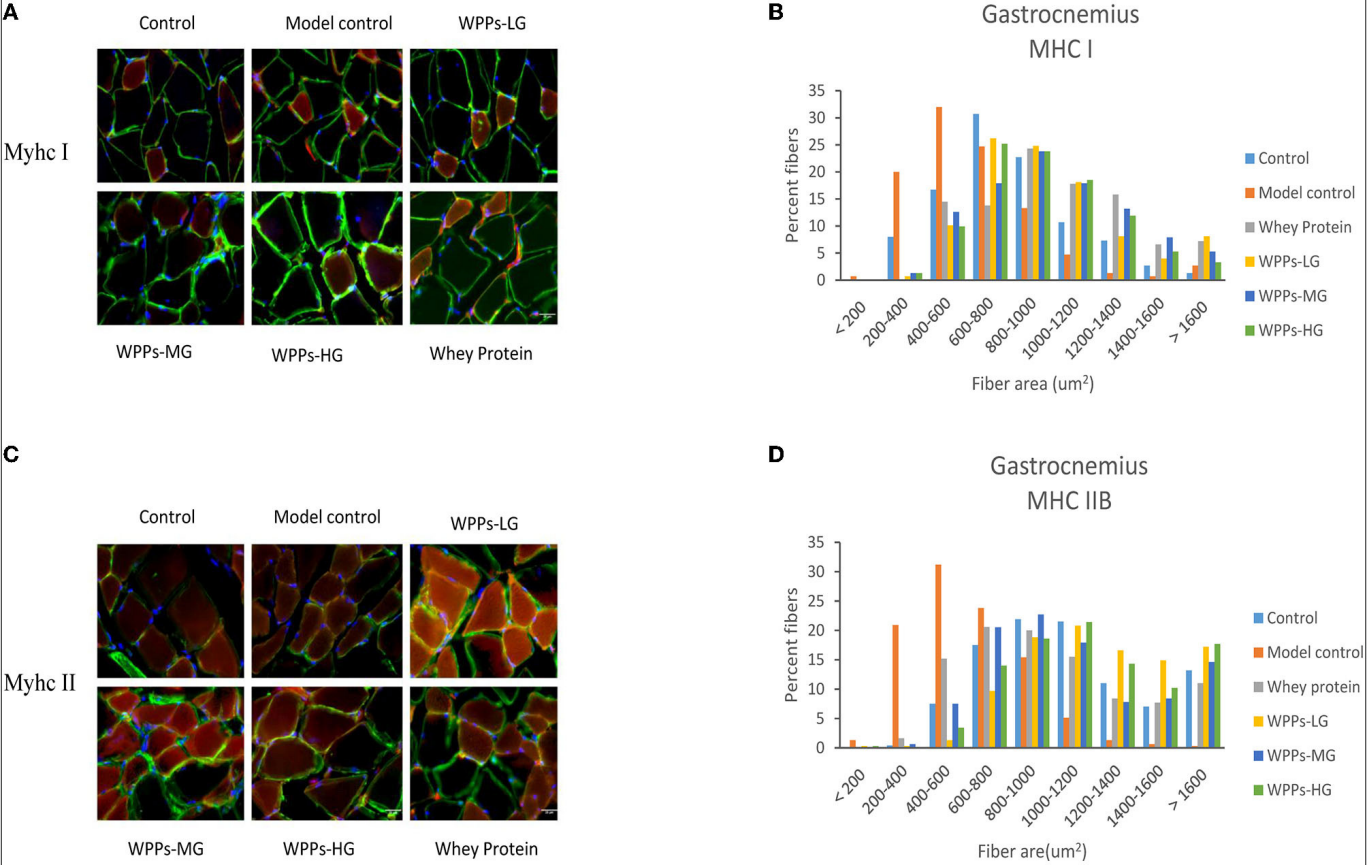
Effects of WPPs on MyHC expression in mice (*n* = 3). Representative images and quantification of laminin, MyHC I, and MyHC IIb immunofluorescent staining (red) in gastrocnemius (*n* = 3) **(A,C)**. CSA distribution of MHC IIB fibers (gastrocnemius) and MHC I fibers (gastrocnemius) presented as frequency histograms **(B,D)**.

We next investigated whether WPPs also influenced muscle fiber size. As shown in Figures 5A,B, it can be demonstrated that WPPs increased MyHC I slow-twitch fiber cross-sectional area (CSA). As shown in Figures 5C,D, it can be demonstrated that WPPs also increased MyHC IIb fast-twitch fiber CSA. These results indicate that WPPs increase the CSA of GAS in skeletal muscle.

### Effects of WPPs on the expression of sarcopenia-related proteins in skeletal muscle of mice

**Figure 6:**
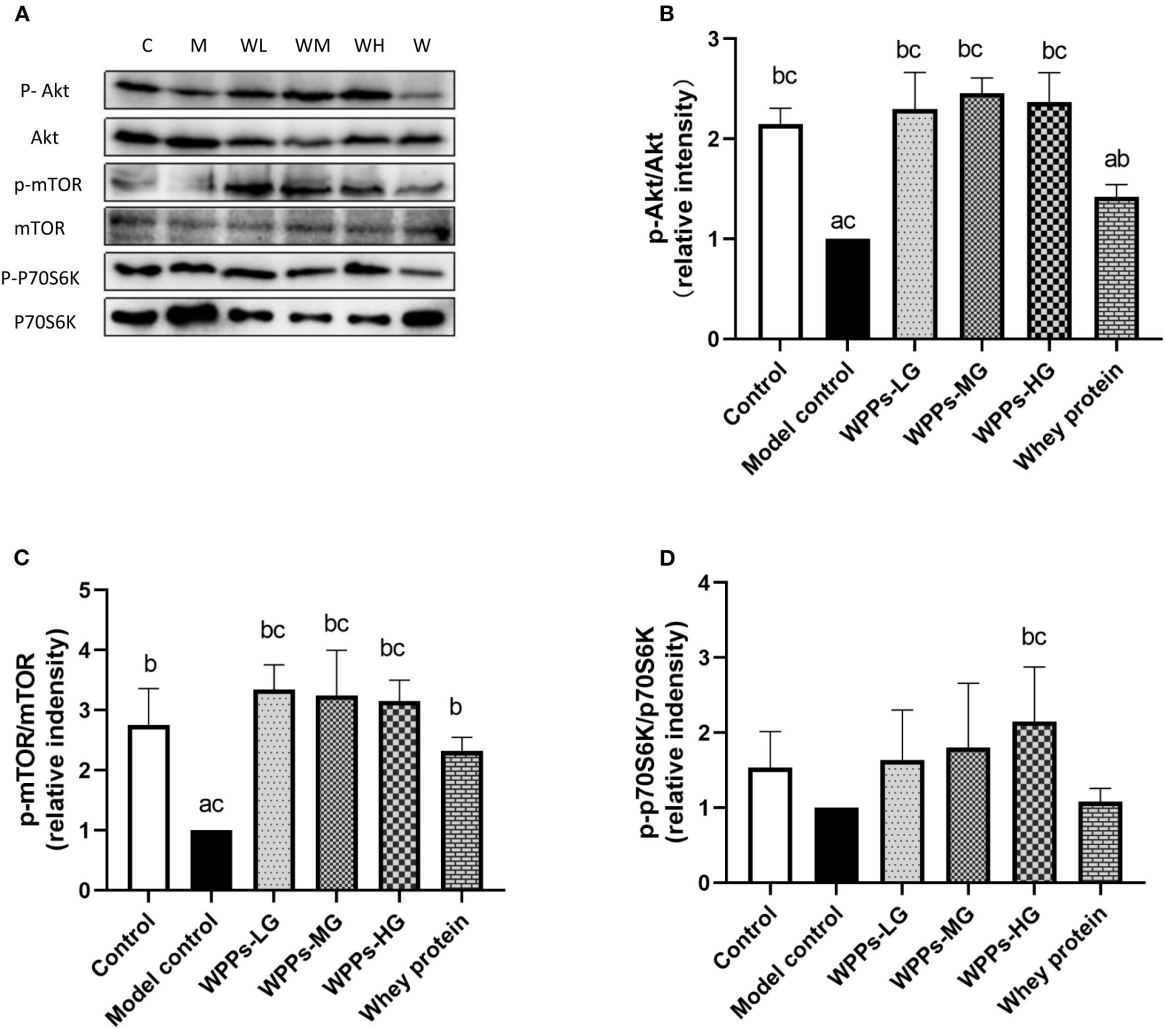
Regulation of mTOR signaling by WPPs in elder mice. Related proteins were analyzed by Western blot **(A)**. Effects of WPPs on Phosphorylation of Akt /Akt in the GAS of mice **(B)**. Effects of WPPs on Phosphorylation of mTOR / mTOR in the GAS of mice **(C)**. Effects of WPPs on Phosphorylation of p70S6K / p70S6K in the GAS of mice **(D)**. Results are expressed as mean ± SD (n = 3). ^a^ p < 0.05 indicates significant difference vs. the control group; ^b^ p < 0.05 indicates significant difference vs. the model group. Whey protein group at a dose of 1.5 g/kg; WPPs-LG, whey protein peptides low dose group at a dose of 0.3 g/kg; WPPs-MG, whey protein peptides medium dose group at a dose of 1.5 g/kg; WPPs-HG, whey protein peptides high dose group at a dose of 3.0 g/kg.

To further investigate the mechanism of WPPs on the sarcopenia, sarcopenia-related proteins were detected. As shown in Figure 6, we assayed the effects of WPPs on the phosphorylation status of Akt. The result showed that WPPs increased the phosphorylation of Akt, Moreover, WPPs exert better effects than whey protein (Figure 6B). The activation of mTOR (Figure 6C) showed that WPPs and whey protein were able to significantly increase the phosphorylation of mTOR, and WPPs had better effects than whey protein. Then, the phosphorylation status of key downstream genes of mTOR was detected. As shown in Figure 6D, the addition of WPPs-HG increased P70S6K phosphorylation in Thr389. These results suggest that WPPs can promote protein synthesis through Akt/mTOR signaling pathway.

## Discussion

Sarcopenia is a hallmark of aging, and it can bring all kinds of negative outcomes. In the present study, the aging mice were induced by d-gal *via* intraperitoneal injection. D -gal-induced aging animal models have been extensively used to study aging mechanisms (27). Our research suggests that the WPPs may be an effective intervention substance for sarcopenia. The results show that WPPs can significantly improve the muscle loss and promote physical performance in aging mice. Specific mechanisms include increasing the level of IGF-1 and promoting protein synthesis *via* mTOR signaling. All the above prove that WPPs has the potential to improve sarcopenia. Moreover, it suggested that WPPs might be a better alternative supplementation in sarcopenia, compared with the whey protein.

Aged rodents (rats or mice, over 24 months) are regarded as suitable model study the sarcopenia, corresponding to human elderly (older than 60 years old) (31, 32). In the previous study, the aging mice were constructed by d-gal, whose aging degree of model mice were certified to meet normal aging standards including physiological and biochemical characteristics (22). Additionally, in recent years, it was revealed that the muscle mass/body mass ratio, cross-sectional area, and fiber diameter obviously reduced in the d-gal induced aging model. The literatures showed that d-gal induced model could be used in the study of age-dependent skeletal muscle degeneration [29.30]. We also found d- gal-induced aging mice had similar aging performance in our study.

Sarcopenia is typically manifested as the decline of muscle mass, strength, and physical performance. In this study, Muscle mass was assessed by calculating body composition and wet weight of four muscles (GAS, SOL, TA and EDL). Meanwhile, the muscle weight/body weight ratio was calculated. Compared with the model control group, the data showed that lean/body (%) and muscle wet weight significantly increased in WPPs groups. In addition, it indicated that WPPs supplementation had the potential to slow down the loss of muscle mass, and significantly provoke an increase in the weight of GAS and EDL wet weight as well as EDL/body weight. To assess the functional consequences of skeletal muscle loss, the grip strength and the hanging wire test were used to evaluate the muscle strength of the d-gal-induced aging mice after WPPs intervention. Previous studies have shown that maximal muscle grip strength can be improved by replacing casein protein with the whey protein in the diet, but not muscle mass (13, 33). Similarly, there was no difference between the whey protein and the model control group in muscle mass. Moreover, there was no difference between the whey protein and model control group in grip strength, grip strength/ body weight, and the time of latency to fall. However, WPPs could significantly increase the grip strength and grip strength/ body weight, compared with model control group and the whey protein, according to our research finding. Although there were no markable differences on the time of latency to fall among groups, it can be found that the mice in WPPs group fall later than that in the whey protein and model control group. The mass and physical performance results suggest that WPPs has the potential to improve aging-related muscle disorders.

Sarcopenia is characterized by a progressive reduction of the number and size of myofiber, loss of motor units, and a shift in fiber-type composition (34). Compared with type I, type II was the predominantly affected muscle fiber in sarcopenia. In this study, the CSA, and the ratio of type I (slow twitch) and II (fast twitch) fibers were identified by IHC staining under confocal, in GAS. The result of fiber morphology indicated that WPPs attenuated sarcopenia with a greater CSA both in the type I and IIB fiber in this study. As a hallmark of sarcopenia progression, the preferential atrophy of type IIB fiber is occurring with aging. Moreover, in our study, the type IIB fiber area was significantly reduced in the model control, and WPPs supplement could effectively ameliorate it. Meanwhile, type I fibers area, known as slow fibers, were also improved in WPPs treated mice. Our results indicated that the beneficial effect of WPPs is achieved by increasing the CSA of muscle fibers.

As a substantially reflection on regulation of muscle mass and fiber size, disrupted protein homeostasis contributes to one of the mechanisms in initial process. And improving protein synthesis was a putative strategy to slow down the progression of sarcopenia. IGF-1/Akt/mTOR signaling was known as one of the critical pathways in muscle protein synthesis (35). The level of IGF-1 is lower inserum of sarcopenia patients (6, 34). In muscle, IGF-1, a regulator of muscle mass and hypertrophy, activates Akt leading to mTOR phosphorylation. The phosphorylation of Akt in muscles, resulting in the upregulation of the phosphorylation of p70S6K *via* the mTOR phosphorylation; these are the key regulators of protein synthesis in muscles (36, 37). mTOR is the main regulator in keeping muscle mass through protein synthesis because it controls the balance between metabolic and catabolic processes. Therefore, the Akt/mTOR pathway is a core module to maintain muscle in aged skeletal muscles (38). And the responsiveness of Akt/mTOR signaling, maybe as a trigger, is diminished during aging (39, 40). The prior studies highlighted the advantages of the whey protein, as well as BCAA, which could suppress atrophy *via* the Akt/mTOR signaling cascade (13). Thus, we chose IGF-1/Akt/mTOR pathway, to decipher the mechanism of action behind the beneficial effect of WPPs. In the present study, we found that WPPs activated Akt, mTOR, and p70S6K signaling molecules with elevated phosphorylation, when compared with the model control group and the whey protein. It suggested that WPPs might promote protein synthesis through Akt/mTOR signaling pathway.

The increments of inflammation and oxidative stress would induce the alterations in DNA, lipids, and proteins, which may contribute to the reduction of muscle protein synthesis and mitochondrial dysfunction in the elderly (6, 33, 41). High levels of proinflammatory cytokines were associated with the loss of muscle mass and poor physical function in older adults. Therefore, age-related chronic low-grade inflammation could be a vital contributor of sarcopenia (42). We demonstrated that WPPs could reduce the level of TNF-α, IL-1 and IL-6 in serum of aging mice (43, 44). These findings are relevant to the elderly and those who are frail or suffer from inflammation and loss of muscle quality. Additionally, studies have confirmed that increasing the intake of antioxidant and anti-inflammatory properties components in diet or in supplementation style could effectively prevent the negative impact of antioxidant deficiency, including oxidative status, lower muscle strength and muscle fatigue after an exercise challenge (10, 19, 45–47). As a typical bioactive peptide, WPPs has been shown to have great anti-inflammation and antioxidation biological activities (22–24). Hence, it indicated that WPPs might attenuate sarcopenia *in vivo* through two distinct mechanisms: by activation of Akt/mTOR signaling pathway and by reduction of inflammation levels and oxidative stress associated with aging.

Our study had several limitations. On the one hand, the dynamic intervention effect is still unclear as the results are of only one time point of sampling. On the other hand, although the results of this study provided, *in vivo*, the direct results of differences in availability between WPPs and the whey protein on sarcopenia, how to do the dose extrapolation is still not clear. Therefore, clinical trials and primary cell culture should be carried out further, which will be helpful in confirming the observed effects, deep interpretation of the mechanism, as well as human applications.

## Conclusion

In conclusion, the results of this experiment indicate that WPPs can improve muscle loss in d- gal-induced aging model under the conditions of this experiment. WPPs promote muscle protein synthesis in the aged skeletal muscle, targeting mTOR phosphorylation. Additionally, we have demonstrated that supplementation of WPPs was a more effective measure to attenuate the skeleton muscle disorder *in vivo* for sarcopenia in comparison with the whey protein. Thus, it suggests that WPPs supplementation is an effective and optimal strategy for sarcopenia prevention and treatment.

## Data availability statement

The original contributions presented in the study are included in the article. Further data and material inquiries can be directed to the corresponding author.

## Ethics statement

The animal study was reviewed and approved by Peking University Animal Research Committee.

## Author contributions

XW and MX conceived and designed the study and wrote and revised the article. XW, XY, and NZ performed the experiments. XW analyzed the data. MX and YL was responsible for conceptualization, methodology, and supervision. The published version of the manuscript has been approved by all authors.

## Conflict of interest

The authors declare that the research was conducted in the absence of any commercial or financial relationships that could be construed as a potential conflict of interest.

## Publisher's note

All claims expressed in this article are solely those of the authors and do not necessarily represent those of their affiliated organizations, or those of the publisher, the editors and the reviewers. Any product that may be evaluated in this article, or claim that may be made by its manufacturer, is not guaranteed or endorsed by the publisher.
